# Assessment of Tear Film Quality among Smokers Using Tear Ferning Patterns

**DOI:** 10.1155/2016/8154315

**Published:** 2016-11-24

**Authors:** Ali M. Masmali, Adil Al-Shehri, Saud A. Alanazi, Ali Abusharaha, Raied Fagehi, Gamal A. El-Hiti

**Affiliations:** Cornea Research Chair, Department of Optometry, College of Applied Medical Sciences, King Saud University, P.O. Box 10219, Riyadh 11433, Saudi Arabia

## Abstract

*Purpose*. To investigate the effect of cigarette smoking on the ocular tear film.* Methods*. Thirty healthy young male cigarette smokers (20–38 years old) and 30 healthy age matched nonsmokers were enrolled in the study. McMonnies questionnaire, slit lamp, and PRT test were used to screen the subjects. Tear samples were collected from the right eyes and tear ferning patterns were observed and graded.* Results*. The mean MacMonnies scores and TF grades were significantly higher in the smoker subjects (mean ± SD = 9.83 ± 5.22 and 0.96 ± 0.54, resp.) compared to nonsmokers (mean ± SD = 5.96 ± 3.06 and 0.41 ± 0.38, resp.). The mean values obtained from PRT and TBUT tests were 22.23 ± 6.35 mm and 12.17 ± 3.81 s for smokers and 22.16 ± 5.63 mm and 14.13 ± 2.62 s for nonsmokers, respectively. Strong correlations were found between MacMonnies scores and both PRT (*r* = 0.596) and TF (*r* = 0.516). There was statistically significant difference in TF grades (*p* = 0.00), TBUT (*p* = 0.036) and McMonnies (*p* = 0.02) between smokers and nonsmokers.* Conclusion*. Cigarette smoking could have a significant effect on the tear film quality of the eye.

## 1. Introduction

Eye dryness is characterized by a reduction in tear volume and stability and increase in tear osmolarity and water evaporation [1, 2]. Visual disturbance, discomfort, blurriness, and damage to the ocular surface are the most common symptoms associated with dry eye [3]. The dryness of the eye can be detected by the use of a combination of different tests to measure the quantity and quality of tears [2]. For example, Schirmer's test [4, 5], phenol red thread (PRT) test [5], tear meniscus height [6], tear osmolarity [7], tear break-up time (TBUT) [8], and tear ferning (TF) test [9]. It has been reported that tear ferning test has the potential to evaluate the ocular tear film and to detect the ocular dryness and has features to be used simply in the clinic and it is useful to investigate the components of tear film [10–12]. Rolando has developed a four-point TF grading scale that classifies eyes as normal (types I and II) and dry (types III and IV) [9]. Masmali et al. developed a five-point TF grading scale to detect the eye dryness [10, 11]. The newly developed TF grading scale can be performed quickly and efficiently within eye clinic. Also, it can differentiate between grades efficiently and its validity and repeatability have been tested [12, 13].

Cigarettes contain many toxic chemicals such as hydrocarbons, aldehydes, nitrosamines, methanol, carbon monoxide, and heavy metals [14]. Such chemicals can cause permanent damage to proteins, lipids, and nucleic acids [15]. Also, cigarette smoking is associated with malignant, respiratory, and cardiovascular life threatening diseases [16]. Moreover, smoking reduces the blood flow which aids blood clots formation within the ocular blood capillaries [17]. Such clots prevent the flow of essential nutrients needed to keep the eye healthy [17]. Annually, more than five trillion cigarettes are smoked which lead to the death of more than five million people [18].

Smoking is a risk factor for many ocular disorders [19–21]. For example, diabetic retinopathy, optic neuritis, cataract, and glaucoma are the most common eye diseases associated with cigarette smoking [19–21]. Moreover, cataract, hypermetropia, and dry eye diseases are associated with passive smoking [22–24]. Several studies have been conducted to investigate the effect of cigarette smoking on the ocular surface and tear film [25–30]. For example, a 5-year study showed that cigarette smokers were more likely to have dry eye by a factor of 1.44 compared to sex and age-matched nonsmokers [21]. Also, it was found that participants who quit smoking were more likely to suffer from a dry eye symptom by a factor of 1.22 compared to nonsmokers [21]. Another study suggested that the risk for dry eye syndrome could be increased by twofold as a result of cigarette smoking [30]. On the other hand, the Blue Mountains eye study indicated that cigarette smoking could protect the eye from dryness with an odd ratio of 0.7 [20].

Therefore, the current study investigates the effect of cigarette smoking on the ocular tear film using tear ferning test along with McMonnies questionnaire and PRT test. To the best of our knowledge, there was no previous report where TF test was used to investigate the effect of cigarettes smoking on the quality of ocular tear film.

## 2. Subjects and Methods

All participants signed informed consent prior to the commencement of the research. Ethical approval has been obtained from the College of Applied Medical Sciences Ethics Committee, King Saud University. Subjects were treated in accordance with the tenets of the declaration of Helsinki [31]. Measurements were carried out by the same examiner under normal conditions. Abnormalities within the eyelids, eyelashes, conjunctiva, cornea, and iris were examined using a slit-lamp (a Haag-Streit BX9000 biomicroscope, Haag-Streit, Köniz, Switzerland). All participants completed McMonnies dry eye symptoms questionnaire and the scores were recorded. Dry eye was diagnosed for a score more than 14.5 [32].

Thirty male smokers who ranged in age from 20 to 38 years (mean ± SD = 29.80 ± 5.07 years) and 30 age-matched nonsmokers (mean ± SD = 31.00 ± 7.84 years) were recruited. All subjects were healthy and have no ocular diseases and did not wear contact lenses. The exclusion criteria include youth (less than 20 years), subjects older than 38 years old, and subjects who recently had eye lubricants, ocular surgery, or medications. The subjects smoke on a daily basis, at least one cigarette a day. The subjects were divided into four groups according to duration of smoking (DS) as group 1 (*N* = 9; DS = 1–5 years), group 2 (*N* = 9; DS = 6–10 years), group 3 (*N* = 6; DS = 11–15 years), and group 4 (*N* = 6; DS = 16–20 years). A tear sample was collected first for tear ferning test followed by PRT and TBUT test. A ten minutes' break was applied between tests.

PRT strips (Zone-Quick; Showa Yakuhin Kako Co., Ltd., Tokyo, Japan) with phenol red pH indicator were used. The third changes color, from yellow to light red, when in contact with tears. A 3 mm length of the thread was folded and inserted one-third of the distance from the temporal canthus of the lower eyelid, with the eye in the primary position. After 15 seconds, the thread was gently removed and the red-colored portion was measured (mm) [4, 5]. Dry eye is defined for readings less than 10 mm.

TBUT test was carried out using Pro Glo Fluorescein Strips (Eye Care and Cure, AZ). The test was performed in both eyes three times and the average time was recorded. The patient was asked not to blink while the tear film is observed under a broad beam of cobalt blue illumination. The time in seconds that elapse between the last blink and the appearance of the first dry spot in the tear film was recorded. Dry eye is defined for measurements less than 10 seconds [33].

Tear samples (1 *μ*L) were collected from the lower meniscus of the right eye using glass capillary tubes (10 *μ*L, Sigma-Aldrich Chemical Company, UK). The tears were allowed to dry on clean, unused glass slides for 10 min under normal conditions (room temperature at around 23°C and humidity at no more than 40%). The slides were immediately observed under a digital microscope (Olympus DP72, Tokyo, Japan) with 10x magnification. The ferning patterns obtained were graded according to Masmali TF grading scale using increments of 0.1, in which TF grade less than 2 is considered as a normal eye [10].

## 3. Statistical Analysis

Data were collected using Excel (Microsoft Office 2010, Microsoft Corp., Redmond, WA). SPSS software (IBM Software, version 22) was used to analyze the data. The data were found to be normally distributed (Kolmogorov-Smirnov test, *p* > 0.05) where mean ± SD was used. The relationships between the obtained data (McMonnies, PRT, TBUT, and TF grades) were investigated by using Pearson correlation coefficient. Correlation coefficients were described as small (0.10–0.29), medium (0.30–0.49), and strong (0.50–1.00) [34]. TBUT and PRT tests were applied to both eyes and there were no significant differences in measurements (paired sample* t*-test, *p* > 0.05) between two eyes. Since the data from both eyes are highly correlated, therefore, the measurements from the right eye were used [35].

## 4. Results

Thirty male smokers and thirty age-matched nonsmokers participated in the study. All subjects were healthy and their ages varied between 20 and 38 years (mean ± SD = 29.80 ± 5.07 years for smokers and 31.00 ± 7.84 years for nonsmokers). The mean MacMonnies scores and TF grades were significantly higher in the smoker subjects (mean ± SD = 9.83 ± 5.22 and 0.96 ± 0.54, resp.) compared to nonsmokers (mean ± SD = 5.96 ± 3.06 and 0.41 ± 0.38, resp.). The mean values obtained from PRT and TBUT tests were 22.23 ± 6.35 mm and 12.17 ± 3.81 s for smokers and 22.16 ± 5.63 mm and 14.13 ± 2.62 s for nonsmokers, respectively (Table 1).

**Figure 1:**
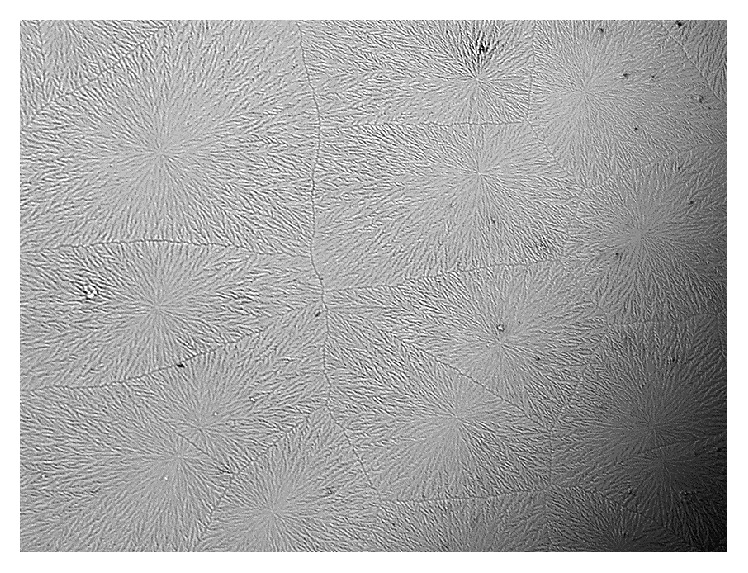
TF pattern obtained from a normal eye smoker.

**Figure 2:**
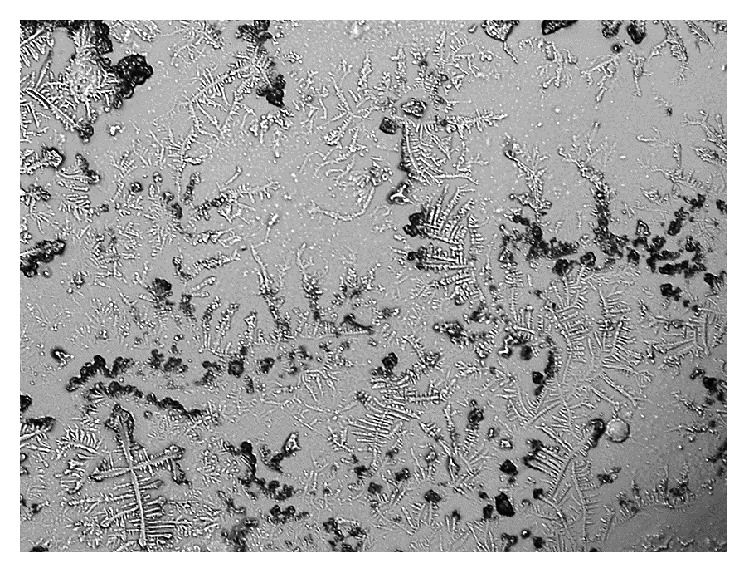
TF pattern obtained from a dry eye smoker.

The overall result showed dry eye symptoms within eight subjects (26.7%) based on McMonnies scores, and three subjects (10%) have lost the tear quality based on both TBUT readings and TF grades. However, none of the subjects showed any changes in tear quantity according to PRT test. Samples for TF patterns for smoker subjects are shown in Figure 1 (normal eye) and Figure 2 (dry eye).

The smoker subjects were divided into four groups according to DS. The first group has 9 smokers (DS = 1–5), the second group has 9 smokers (DS = 6–10), the third group has 6 smokers (DS = 11–15), and the fourth group has 6 smokers (DS = 16–20). McMonnies scores indicated that two subjects (22.2%) in group 2, two subjects (33.4%) in group 3, and four subjects (66.7%) in group 4 have dry eye. TBUT test showed that one subject (16.7%) in group 3 and two subjects (33.4%) in group 4 have dry eye. Based on TF grades, dryness of the eye has been detected in one subject in group 1 (11.1%) and two subjects in group 2 (22.2%).

Duration of smoking (Table 2), in years, was found to have a medium negative correlation (*r* = −0.413) with TBUT, a negative small correlation with TF (*r* = −0.258), small positive correlations with MacMonnies scores (*r* = 0.288), and a small negative correlation with PRT (*r* = −0.197). Strong correlations were found between MacMonnies scores and both PRT (*r* = 0.596) and TF (*r* = 0.516). Correlations among various tests for smokers were found to be small or medium.

For nonsmoker subjects, the correlations among various tests were found to be small or negligible. The correlation between McMonnies questionnaire scores, PRT, TBUT, and TF grades within nonsmoker subjects is reported in Table 3. There were statistically significant differences in McMonnies questionnaire, TBUT and TF grades between smokers and nonsmokers (Mann–Whitney test, *p* = 0.02, 0.036, and 0.00, resp.).

## 5. Discussion

Cigarette smoking is a risk factor for various ocular diseases, one of which is dryness of the eye [27–29]. The most common symptom in cigarette smokers or those exposed to active or passive cigarette smoke is the irritation of eye [36].

The results of the current study are consistent with most of the previous studies. There were significant differences between smokers and nonsmokers according to tear ferning test, McMonnies questionnaire, and TBUT test. The mean values for McMonnies, tear ferning, and TBUT tests in smokers compared to nonsmokers indicate that smoking has effect on the ocular tear film. However, the PRT test suggested no significant differences between smokers and nonsmokers tear volume. The reason for that could be due to the variation in tests in which each test detects a specific characteristic within the tears. The tear ferning patterns (Figures 1 and 2) are clearly showing the differences in tear quality between dry and normal tear samples among smokers and nonsmokers. The tear ferning test is a useful clinical test that can evaluate the quality of tear film.

The mean value for dry eye questioner scores was found to be significantly higher with smokers (6.0 ± 1.17) compared to nonsmokers (2.9 ± 1.80) [28]. Schirmer 1 test showed an increase in tear volume within 44 chronic smokers (30.3 ± 16.7 mm) compared to 37 nonsmokers (23.8 ± 12.4) [29]. Similar results were obtained from a cross-sectional study conducted within 49 chronic smokers [25]. The means for Schirmer scores were significantly lower in smokers (13.30 ± 4.63 mm) compared to those obtained from 53 aged matched nonsmokers (15.45 ± 4.11 mm) [25]. Excessive tearing could be obtained as a result of cigarette smoking [29]. Also, reflex tears would be produced due to instability within tear film [37]. However, the mean value for Schirmer I test within 60 smokers (10.23 mm) was found to be similar to those obtained from 34 healthy nonsmoker subjects (10.65) [28]. Other studies indicated that the results obtained from Schirmer's II test result were not conclusive in which there was no significant differences between smokers and nonsmokers [26, 27]. It appears that the direct link between smoking and dryness of the eye is not well established using Schirmer test.

A study conducted on 51 smokers showed that the mean for TBUT was significantly lower (7.26 ± 1.86 s) compared to 50 age-matched nonsmokers (11.28 ± 1.27 s) [26]. In a cross-sectional study which involves 49 chronic smokers, the means for TBUT were significantly lower (8.24 ± 2.39 s) compared to those obtained from 53 aged-matched nonsmokers (11.15 ± 1.94 s) [25]. Similarly, the mean values for TBUT within 44 smokers were found to be lower (11.9 ± 5.8 s) compared to 37 nonsmokers (14.9 ± 5.5 s) [29]. In addition, similar results were obtained from TBUT test conducted within 29 smokers (7.7 ± 2.7 s) and 26 nonsmokers (9.6 ± 3.1 s) [28]. Even more significant differences in the TBUT mean values were reported in 60 smokers (5.4 s) and 34 nonsmokers (11.2 s) [28]. Also, the mean for TBUT for 15 chronic smokers was much lower (3.2 ± 0.7 s) compared to that obtained for 20 nonsmokers control subjects (14.2 ± 2.4 s) [27]. The decreases in TBUT values within smoker subjects could be attributed to irritation of eye caused by cigarette smoking [38].

Cigarette smoking could to have an effect on the dryness of the eye. Moreover, smoking is very dangerous and causes various diseases, some of which could lead to death. Therefore, all types of tobacco smoking should be avoided and prohibited in public places and in premises to avoid negative impact on nonsmokers and to keep the environment clean.

## 6. Conclusions

There was a significant difference between smokers and nonsmokers based on various tests, which reflects the effect of smoking on the quality of tear film. Further detailed studies are still needed to determine precisely the effect of cigarette smoking on the ocular surface. Also, a larger sample and other tear film evaluation tests (e.g., tear osmolarity) are needed for future studies.

## Figures and Tables

**Table 1:** The mean ± SD for MacMonnies scores, PRT test, TBUT test, and TF grades for smoker and nonsmoker subjects.

Test	Smoker (*n* = 30)	Nonsmokers (*n* = 30)
MacMonnies scores	9.83 ± 5.22	5.96 ± 3.06
PRT	22.23 ± 6.35 mm	22.16 ± 5.63 mm
TBUT	12.17 ± 3.81 s	14.13 ± 2.62 s
TF grades	0.96 ± 0.54	0.42 ± 0.38

**Table 2:** Correlation between McMonnies questionnaire scores, PRT, TBUT, TF grades, and duration of smoking within smoker subjects (*N* = 30).

Test/correlation		McMonnies	PRT	TBUT	TF	DS
McMonnies	PC	1	0.101	−0.254	−0.123	0.288
	Sig.	—	0.596	0.175	0.516	0.123
PRT	PC	0.101	1	0.323	−0.302	−0.197
	Sig.	0.596	—	0.082	0.105	0.297
TBUT	PC	−0.254	0.323	1	−0.272	−0.413^a^
	Sig.	0.175	0.082	—	0.146	0.023
TF	PC	−0.123	−0.302	−0.272	1	−0.258
	Sig.	0.516	0.105	0.146	—	0.168
DS	PC	0.288	0.197	−0.413^a^	−0.258	1
	Sig.	0.123	0.297	0.023	0.168	—

PC Pearson correlation, DS: duration of smoking, and Sig.: significance (2-tailed).

^a^Correlation is significant at the 0.01 level (2-tailed).

**Table 3:** Correlation between McMonnies questionnaire scores, PRT, TBUT, and TF grades within nonsmoker subjects (*N* = 30).

Test/correlation		McMonnies	PRT	TBUT	TF
McMonnies	PC	1	−0.070	−0.274	0.195
	Sig.	—	0.715	0.143	0.301
PRT	PC	−0.070	1	0.001	−0.095
	Sig.	0.715	—	0.997	0.616
TBUT	PC	−0.274	0.001	1	−0.187
	Sig.	0.143	0.997	—	0.323
TF	PC	0.195	−0.095	−0.187	1
	Sig.	0.301	0.616	0.323	—

PC: Pearson correlation, Sig.: significance (2-tailed).
